# Degradation of RC Columns under Combined Exposure to Axial Loading, Stray Currents, and Chloride Ingress

**DOI:** 10.3390/ma17061295

**Published:** 2024-03-11

**Authors:** Igor Lapiro, Rami Eid, Konstantin Kovler

**Affiliations:** 1 National Building Research Institute, Faculty of Civil and Environmental Engineering, Technion—Israel Institute of Technology, Haifa 3200003, Israel; igorlapiro@campus.technion.ac.il (I.L.); cvrkost@technion.ac.il (K.K.); 2 Department of Civil Engineering, Braude College of Engineering, Karmiel 2161002, Israel

**Keywords:** steel reinforced concrete, columns, mechanical loading, chloride transport, stray current

## Abstract

Coastal regions, home to a significant portion of the world’s population, confront a formidable challenge: the corrosive impact of chloride-rich environments on vital infrastructure. These areas often host essential transportation systems, such as trains and metros, reliant on pre-existing electrical infrastructure. Unfortunately, complete isolation of this infrastructure is rarely feasible, resulting in the emergence of stray currents and electrical potentials that expedite corrosion processes. When coupled with conducive mediums facilitating chemical electrocell formation, the corrosion of reinforced concrete elements accelerates significantly. To combat this issue, international standards have been established, primarily focusing on augmenting the thickness of reinforcement bar covers and restricting stray voltage between rails and the ground. Nevertheless, these measures only provide partial solutions. When subjected to service loads, these elements develop cracks, especially when exposed to stray currents and chlorides, dramatically increasing corrosion rates. Corrosion products, which expand in volume compared to steel, exert internal forces that widen cracks, hastening the deterioration of structural elements. The study deals with the degradation of reinforced concrete columns under the combined action of loads, chloride-rich environments, and electrical voltage-simulating stray currents. In these conditions, degradation and reduction of load-bearing capacity accelerate compared to unloaded conditions, significantly amplifying the corrosion rate. Astonishingly, even in the absence of mechanical loads, stray currents alone induce tensile stresses in elements due to corrosion product formation, leading to longitudinal cracks parallel to the reinforcement bars.

## 1. Introduction

The first electric train line in the United States began operating in 1888 in Richmond, Virginia. Almost immediately, corrosion problems were observed in the infrastructure, including damage to the rails and spikes. Further investigation revealed that current leakage from the rails was the primary cause of this corrosion [1]. DC-powered transit systems have had to undertake significant repairs and modifications to their signal and traction power systems due to inadequate negative return rail isolation. The Institute of Electrical and Electronics Engineers (IEEE) published a research report in the 1990s that highlighted the considerable financial costs of this issue. The report estimated that a significant portion of the $500 million per year in losses from stray current corrosion was incurred by DC-powered transit properties and the surrounding infrastructure assets [2]. Most of the world’s population lives in the coastline area, and thus most infrastructures are exposed to an environment containing a critical amount of chlorides, which in time damages steel components [3,4,5]. Not only steel but also reinforced concrete elements can be damaged by stray currents or the presence of chlorides.

A study that examined the effect of stray currents on the calcium leaching of cement-based materials reached the following conclusions: The compressive strength of cement-based materials decreases the most, which is 13.7% lower than that of specimens corroded under 0 V [6]. Stray currents can cause a migration effect that accelerates the ingress rate of SO^−2^_4_ ions, leading to a severe external sulfate attack on cement-based materials. This attack results in the formation of ettringite and gypsum, which ultimately causes cracking and a significant reduction in strength, up to 47.1% [7]. Stray currents can increase chloride penetration and thus obtain the threshold of their critical chloride in a shorter period, which will accelerate the corrosion process and the damages involved [8,9].

International standards EN 50122-2:2010 [10] and ASTM-G165-99-R2012 [11] limit the deviation of electric voltage due to stray current in reinforced concrete elements up to 0.2 V to protect against stray current [2,12]. However, this value is problematic for implementation and monitoring since reinforced concrete has a natural electric potential of this order of magnitude [13]. Electric potential values that can be measured in concrete range from −0.78 to−0.48 vs. SCE. Therefore, in areas that combine stray currents and chlorides, increased damage to reinforced concrete elements is observed.

It is known that electric potential has a critical effect on the formation and sustainability of a passivation layer [14]. The corrosion of steel bars is the main cause of failures in structural elements made of reinforced concrete. Nowadays, it is not possible to completely prevent it but only to slightly delay its development [15]. The partial solution to the protection against the corrosion process of the bars in reinforced concrete is by increasing the thickness of the concrete cover, thus essentially increasing the volume of the alkaline environment in which the steel bar is located and providing a greater protective distance that the carbonation and chloride ions must pass to reach the concrete-bar interface. However, due to the cracks that exist in the concrete, both processes (ingress of chloride ions and the reduction of alkalinity) are accelerated, and thus the thickness of the cover does not provide protection for the element [16].

After the appearance of corrosion, one can expect a loss in the bond strength between the concrete and the steel bar and a reduction in the cross-sectional area of the steel bars. The formation of iron oxide may lead to additional unwanted side effects (such as the expansion of the material), which will lead to increased parallel cracking and even to the detachment of concrete parts from the structural element [17,18].

It was shown that reinforcement corrosion led to a decrease in the final axial load and an increase in both the axial and lateral strain of RC columns without service load. In combination with a service load at the same corrosion values, the ultimate capacity presented higher strength values than the samples without a load in combination with the corrosion of the reinforcing bars [19]. On the other hand, in every combination of corrosion, the values of the axial compressive strength were lower than in reference samples without corrosion [19]. The research results show that the structural integrity of bridges does not change significantly within 25 years of exposure to environmental factors in the absence of stray currents. While corrosion of the bar rarely affects its performance during low-intensity earthquakes, it markedly reduces its capacity to withstand high-intensity earthquakes [20]. Under accelerated conditions of corrosion, this degradation can be even more pronounced. Additionally, studies have shown that non-corroded bridges have a sufficient safety margin against earthquakes, ranging from 35% to 65%, depending on the direction of the seismic force. However, this safety margin drops by 20–40% when the reinforcing bars are corroded and lose 20–30% of their mass [21].

Insufficient column confinement and corrosion of the embedded bars have been identified as some of the problems existing within such structures. Problems have increased factors to adjust existing models for predicting the strengths of reinforced concrete columns and incorrect assessment. The location and direction of the bars influence the rate of corrosion due to stray currents and corrosion in general. It was shown that bars parallel to the stray current have a higher potential for corrosion than those perpendicular to the stray current [22]. In addition, the thickness of the concrete cover has an effect on the corrosion created in steel bars and how it is distributed to the other bars [23]. Any metal that is electrically connected to the concrete or the steel reinforcement will be harmed by stray currents, which will discharge somewhere on their journey back to the source. At the location where the current discharges, corrosion takes place. Installation of resistance bonds is one proven technique for preventing this kind of corrosion, which has been used for many years in the buried pipeline business. Resistance bonding involves electrically connecting the impacted structure to the source of stray current using a resistor. This ensures that the current follows a metallic path back to its source, preventing any metal from the afflicted structure from being lost [1,24]. Galvanic anodes are used in a different technique to direct the collected current. The gathered current is sent into the electrolyte (groundwater), where it is then diverted back to its source from the anode’s surface. Instead of the steel reinforcement corroding in this instance, the galvanic anode does. The current discharge electrode must provide the path of least resistance to the source, so proper system design, installation, and maintenance are crucial [24]. When looking at the standards discussing reinforced concrete design, we cannot find any reference to defense against stray currents. On the other hand, it is possible to find a reference dealing with an environment rich in chloride ions [24,25,26].

Previous studies usually examined accelerated corrosion without addressing the effects of concrete cover thickness under stray current conditions. The effect of stray current on element deformations before loading and crack formation is still not studied as well as the influence of the quantity of steel on corrosion development. The purpose of this study is to try to bridge these gaps and strengthen the knowledge of the mechanisms of reinforcement corrosion in concrete under the combined action of mechanical loading, chloride ingress, and stray current.

## 2. Research Scope and Goals

This study examines the effect of axial loading, chloride ingress, and stray current on the degradation and structural resistance of reinforced concrete columns. In addition, the following research aspects related to physical, mechanical, and chemical mechanisms of such degradation were studied:

- Effect of the serviceability compressive axial load on the crack formation
- Examination of the threshold of chloride concentration
- Effect of the concrete cover thickness

## 3. Experimental Program

**Figure 1:**
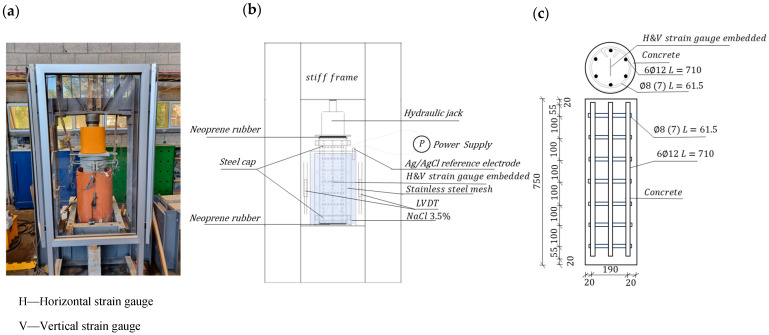
(**a**) Main test setup; (**b**) Instrumentation and measurement devices; (**c**) Reinforcement cage in column specimens (dimensions in mm).

Two experimental setups were carried out, with the main one consisting of 13 reinforced concrete column specimens with a height of 750 mm, a diameter of 230 mm, and a concrete cover of 20 mm (Figure 1c). Three reference samples were subjected only to compressive axial loading up to failure without being exposed to controlled environmental conditions (reference specimens). An additional three specimens were subjected to compressive axial loading up to failure after being exposed for four weeks to a voltage of 9 V and an aggressive environment containing 3.5 percent chlorides (SDFCL, Mumbai, India). The other three specimens were simultaneously exposed to a similar voltage/chloride condition and to a constant compressive axial load corresponding to 60% of the specimens’ ultimate strength (set based on the average experimental ultimate load reached by the three reference samples) before applying the axial loading up to failure. The combined exposure to a chloride solution, a 60% load, and 9 V lasted for 30 days. Three more columns underwent cycles of wetting and drying in salt water based on a commonly used procedure [27,28] (four weeks in the wet state followed by four weeks in the dry state). Finally, a single additional column was kept as a reference over time.

The electrical voltage in these experiments was chosen based on the following considerations: On one hand, the 50,122 standard [10,29] specifies a maximum hazardous voltage of 90 V, taking into account public safety conditions. On the other hand, the voltage that can cause corrosion ranges from 0.85 to 1.2 V, depending on the pH of the concrete, ranging from 9.0 to 13.5, respectively [30]. In addition, the voltage between the rail and reinforcing bars in concrete elements depends on the distance from the rail and the concrete coating’s electrical resistance. This resistance ranges from 50 to 8000 Ωm, while the earth’s electrical resistance ranges from 50 to 3000 Ωm [31]. For an average soil resistance of 30–60 Ωm, the current density decreases by an order of magnitude from its initial value at a distance of 100 m. A similar decrease by another order of magnitude occurs at a distance of 500 m. Beyond 500 m, the current becomes very small [31].

According to safety standards, stray voltages above 10 V must be handled by authorities [32]. This value is close to the highest voltage measured in site conditions, which can reach 10 V [33,34,35]. In the current study, 9 V was chosen, which is equal to 10% of the maximum hazardous voltage and close to the 10 V measured in site conditions and specified by [34].

**Figure 2:**
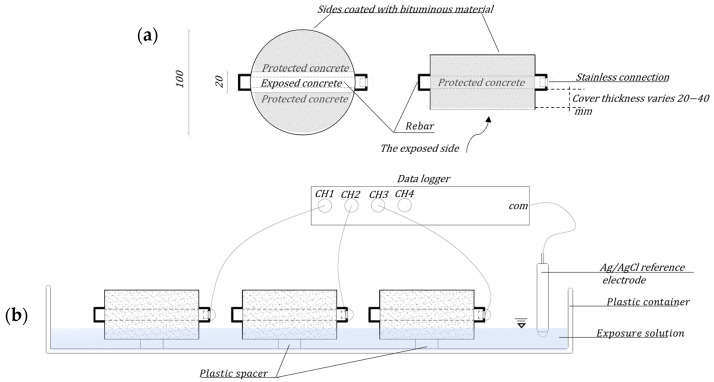
(**a**) Samples for examining the effect of cover thickness and chloride concentration; (**b**) Test setup for examining cover thickness and chloride threshold.

The second experimental setup aims to examine the effect of the chloride concentration, the thickness of the concrete cover, and the amount of steel on the electrochemical potential for corrosion. Concrete disc-shaped samples were prepared with varying cover thickness, containing an embedded steel rod with a diameter of 8 mm and 12 mm. The thickness of the concrete cover was 20, 30, and 40 mm (Figure 2a). A total of 18 samples were tested. The samples had a diameter of 100 mm and varied in height depending on the thickness of the concrete cover. The outer cover of the rods extended to about 20 mm.

### 3.1. Materials

In this study, a concrete mixture was prepared based on CEM I 52.5N cement, coarse limestone aggregate, fine quartz aggregate, and tap water; the proportions can be seen in Table 1.

The steel reinforcement bars were of type B500C, which meets the requirements of the European standard BS EN 10080 [37]. Ribbed longitudinal bars of 12 mm diameter and hoops of 8 mm diameter were used (Figure 1c).

### 3.2. Casting and Curing

The column samples were kept in plastic molds for 24 h in ambient conditions of 24 °C and RH = 65 ± 5% after casting. After this time, the samples were removed from the mold and immersed in water for 90 days. Samples for testing concrete properties included three cylinders with a diameter of 150 mm and a height of 300 mm, three 270 mm × 70 mm × 70 mm prisms, and three 100 mm cubes. The dimensions of the samples were selected based on the requirements of the EN 12390-1 standard [38]. All these samples underwent curing in water with calcium hydroxide until the day of testing. The tests were performed in 7, 28, and 90 days after casting the samples. The disc samples for testing the effect of cover thickness and chloride concentration threshold were kept in water with calcium hydroxide for 90 days and then were sealed with a bituminous coating material (Figure 2a).

### 3.3. Mechanical Properties of Concrete and Steel Reinforcement

**Figure 3:**
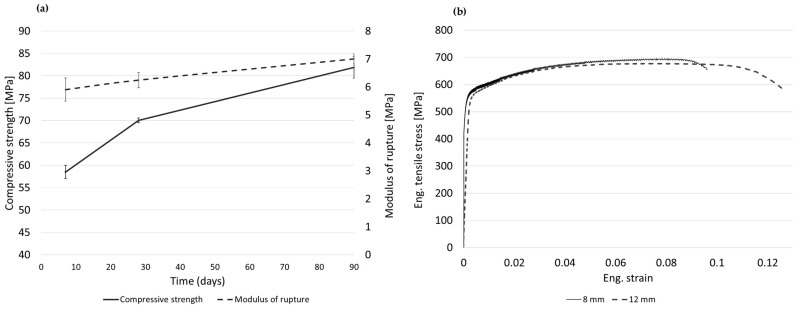
(**a**) Testing results for concrete; (**b**) Testing results for reinforcing steel.

Figure 3a shows the results of the concrete compressive and tensile strength (or the modulus of rupture based on a flexural test) up to 90 days after casting (3 samples were tested in each time period). It can be assumed that the strength of the concrete obtained in 90 days is the representative strength of all types of samples. Figure 3b shows the average results of the tensile stress—strain relationships of 6 reinforcement bars: 3 longitudinal bars with a diameter of 12 mm and 3 bars with a diameter of 8 mm that were used for hoops in the reinforced concrete columns. At 7 days, the samples showed a compressive strength of 59 MPa with a standard deviation of 1.5 MPa. By day 28, the compressive strength increased to 70 MPa with a standard deviation of 0.5 MPa, and by the 90th day, it reached 82 MPa with a standard deviation of 2.4 MPa. Regarding the Modulus of Rupture, the results were 5.9 ± 0.4, 6.2 ± 0.3, and 7.0 ± 0.2 MPa for days 7, 28, and 90, respectively. Additionally, the steel exhibited a strength of 677 and 696 MPa for rebar diameters of 12 and 8 mm, respectively.

### 3.4. Methods

The effect of stray electric voltage was studied by applying an external voltage directly to the reinforcement bars. The positive pole of a voltage source was connected to the reinforcing bars, and the negative pole was connected to a 316-type stainless steel grid. The electrical voltage between a reinforcing bar and a saltwater environment in contact with concrete was measured using an Ag/AgCl reference electrode and a data collection system. The axial compressive load was applied using a hydraulic jack (Figure 1a).

The analysis of the state of corrosion was based on half-cell potential measurements following the guidelines specified in ASTM standard C876 [39]. The measurements in the main experiment setup (i.e., concrete columns) were carried out continuously, with readings recorded every 5 min. The experiment on the disc samples was also performed continuously by receiving data every 5 min. In the experiment that underwent wetting and drying, the measurement was performed once per week.

The open-circuit potential, also referred to as half-cell potential, measures the difference between an electrochemical potential and a reference electrode at various points to generate potential. The assessment of the outcomes was based on the following criteria: (a) when potentials in a particular area exceed −0.20 V Copper Sulphate Electrode (CSE), there is a high probability (above 90%) that there is no corrosion of reinforcing steel at the time of measurement; (b) when potentials in a specific region fall within the range of −0.20 to −0.35 V CSE, the corrosion activity of the reinforcing steel in that area is uncertain; and (c) if potentials in a certain zone are less than −0.35 V CSE, there is a greater than 90% chance that there is corrosion of reinforcing steel in that area at the time of measurement [39,40,41]. During the experiment, the electrical resistance of the samples was tested on the surface according to the AASHTO T 358 guidelines [42].

Figure 2 shows the setup and samples used to determine the chloride threshold value for corrosion. The experiment was performed according to the instructions of the experimental protocol to determine the chloride threshold value for corrosion [43]. After two weeks in a chloride-free solution, the exposure solution was replaced with a solution of 3.5% NaCl by weight. The water used to prepare the solution was deionized. The amount of solution was 10 L and was not changed throughout the experiment. The exposure tank was closed by a plastic cover to limit the evaporation of the solution and associated changes in chloride concentration. During exposure to chloride, the water level was checked daily for all the samples and maintained constant. The potentials of the samples were recorded every 5 min.

The initiation of corrosion in each sample was evaluated under the following two conditions: (1) The potential falls by more than 150 mV from the passive level within a period of 5 days or more; (2) the potential remains stable (for 10 days) at the achieved negative level, with fluctuations of ±50 mV [43]. If both conditions were not met within 60 days, the concentration of NaCl in the solution was increased to 7% by weight. After 120 days, the NaCl concentration in the solution was raised to 10% by weight, and it was maintained at this level thereafter. Any increase in concentration required verification of both conditions [43].

The permeability of chloride ions in the experiment was determined using the AgNO_3_-based colorimetric method. This method involves applying an AgNO_3_ solution exclusively to a recently broken cross-section of concrete. This process results in the creation of distinct white and brown areas, separated by a noticeable boundary with a clear color transition. Typically, the depth of the white zone is considered the extent of chloride penetration, while the brown zone indicates the absence of chloride [44]. The chemical reactions involved in this method are as follows:

1. ${\mathrm{Ag}}^{+}+{\mathrm{Cl}}^{-}\to \mathrm{AgCl}\left(\mathrm{white}\right)$ Chemical reactions chloride-containing zone and chemical reactions in the chloride-free zone
2.
${\mathrm{Ag}}^{+}{\mathrm{OH}}^{-}\to \mathrm{AgOH}\to {\mathrm{Ag}}_{2}O\left(\mathrm{brown}\right)$

By employing this method, only free chlorides, which are responsible for initiating corrosion, can be identified, whereas chemically bound chlorides from the cement compositions remain undetected. Distinguishing between chemically bound chlorine and free chlorine allows for the assessment of structural deterioration risk for the following reasons:

- Reducing the concentration of free chloride near the reinforcing steel will reduce the risk of corrosion;
- Chloride binding will inhibit chloride penetration;

The formation of Friedel’s salt $\left(3\mathrm{CaO}\cdot {\mathrm{Al}}_{2}O_{3}\cdot {\mathrm{CaCl}}_{2}\cdot 10H_{2}O\right)$, resulting from a chemical reaction between chlorides and C_3_A, is characterized by a less porous structure, which slows down the transport of Cl ions [45].

The chloride profile on the section was built using the protocol described in [46,47]. After exposure to chlorides, samples were drilled to investigate the chloride concentration at the interface between the concrete and the reinforcing bar. In the initial step, to assess surface chloride concentration, drilling was performed to a depth of 1 mm, collecting approximately 5 g of powder. Subsequently, drilling proceeded in 2 mm increments to prevent contamination of the desired sample with chlorides from previous layers. Each layer was stored in a sealed, impermeable plastic container until testing to prevent external influences. The drilling continued until the depth of the reinforcing bar was reached.

To examine whether reaction products of NaCl were formed and to verify the chloride profile, XRD (X-ray diffraction) analysis, which is the most common technique for characterizing materials in the literature [48], was applied. The details of the specimen preparation and following XRD analysis were identical to those reported in previous work [49], namely:

- The samples were initially manually ground to achieve a particle size of less than 75 microns.
- The XRD analysis utilized a Malvern Panalytical EMPYREAN X-ray diffractometer under specific measurement conditions: a CuKα1,2 X-ray source (λ = 1.5406 Å), with the X-ray generator operating at 45 kV and 40 mA, and a Goniometer radius of 240 mm. The incident beam optics comprised a ¼° divergence slit, 10 mm mask, 0.04 rad Soller slit, and 1° anti-scatter fixed slit. The diffracted beam optics included an 8 mm anti-scatter fixed slit and a 0.04 rad Soller slit. The detection system utilized a PIXcel 3D detector in a 1D continuous scan mode. Scanning was performed in Brag–Brentano geometry, covering a 2𝜃 range from 10° to 65° with 0.026° steps and a counting time of 84 s/step.
- The XRD data were analyzed using Panalytical HighScore Plus software (version 5.1), referencing ICDD PDF-4 Minerals and PAN-ICSD databases.

For a more comprehensive understanding of the distribution of chlorine ions in the samples, SEM (scanning electron microscope) observations and EDS (X-ray scattering spectroscopy) analysis were performed on sections of the freshly cracked sample. Prior to SEM and EDS tests, the samples underwent pre-treatment. Initially, the samples were coated with carbon and then dried in a vacuum within a carbon hood for 30 min. Subsequently, the vacuum was released using nitrogen, and immediately afterward, the samples were placed into the SEM microscope (FEI E-SEM Quanta 200, FEI Company, Hillsboro, OR, USA) under low vacuum conditions. The SE detector was activated, and the working voltage was set to 20 kV.

## 4. Results

### 4.1. Reinforced Concrete Columns

**Figure 4:**
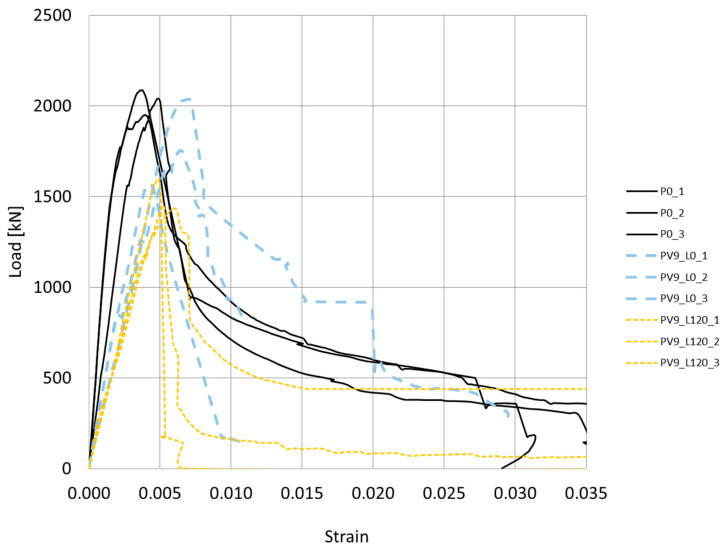
Compressive axial load—strain diagrams of the reference samples (P0_1–3), the samples exposed to a voltage of 9 V (PV9_L0_1–3), and the samples exposed to the combination of service load of 1200 kN and an electrical voltage of 9 V (PV9_L120_1–3).

Figure 4 depicts the axial compressive load versus strain curves for nine reinforced concrete specimens. The maximum measured compressive axial load reached 2122 kN. The subsequent section details the influence of corrosion, both in the presence and absence of a constant axial compressive load (emulating a service load), on the structural response of the reinforced concrete column specimens.

#### 4.1.1. Effect of Corrosion

After subjecting the specimens to a simulated stray voltage of 9 V and testing the axial compressive load-strain behavior, the results were obtained as depicted in Figure 4. The black line curves represent the reference samples, while the blue curves depict the behavior of the samples exposed to the electrical voltage and an aggressive chloride-containing environment. On average, the latter samples exhibited a peak load of 1785.4 kN. This indicates a reduction of 11.85% in the bearing capacity, specifically in terms of compressive strength. Moreover, the displacement ductility factor is defined as $\mu =\varepsilon_{cf50}/\varepsilon_{P}\;$ where $\;\varepsilon_{cf50}$ is the strain after peak load when the force drops to 50% of the maximum peak load and $\varepsilon_{P}\;$ is the concrete compressive strain corresponding to the peak load [50]. For the reference samples, the average ductility factor is  μ=1.94, while for the samples affected by voltage/chloride, it is μ=1.69. This indicates a reduction of 12.8% in ductility. Moreover, for the samples subjected to both voltage/chloride and a service load of 1200 kN, the average measured peak load was 1491 kN, with a calculated ductility factor of μ=1.13. This signifies a decrease of 34.7% and 41.7% in bearing capacity and ductility, respectively.

#### 4.1.2. Effect of Wetting and Drying Cycles

**Figure 5:**
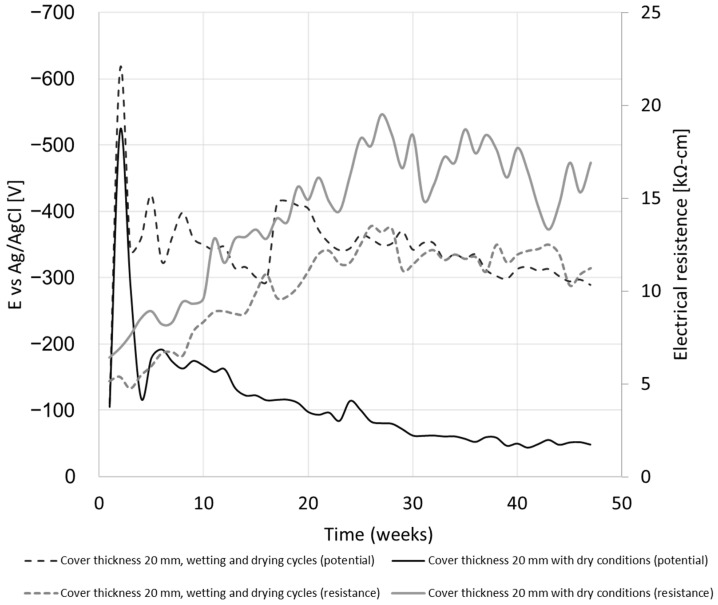
Comparison of half-cell potential and resistance change due to wetting and drying cycles compared to a constant dry state.

The measurement results of the half-cell potential for the samples subjected to wetting and drying under the conditions of a 3.5% chloride solution are compared with a sample in a dry state, devoid of any exposure to chlorides, as shown in Figure 5. The wetting and drying cycles followed the following plan: four weeks in the wet state, followed by four weeks in the dry state. The first measurement was taken after 90 days from the day of casting. After three weeks, an evident electrochemical reaction was observed, leading to an increase in the half-cell values by approximately 620 mV for the exposed specimens and 530 mV for the specimens without chloride exposure. Following this period, an increase in electrical potential becomes noticeable, particularly when comparing the samples subjected to wet and dry cycles against those that have remained consistently dry. The potential difference is approximately 250 mV. The dry samples exhibit a mere 10% probability of undergoing a corrosion process. In contrast, the samples subjected to the cycles display a markedly higher probability of 90% up until week 35. After this point, there is a noticeable decline in the likelihood, of tapering off to a 50% probability of experiencing a corrosion process [39,40,41].

Measurements of electrical resistance on a surface can be used to estimate the permeability of chlorine ions. According to Figure 5, the values obtained in the first weeks were lower than the values stated in the AASHTO TP 95 guidelines. These guidelines indicate that values smaller than 12 kΩ-cm (or 9.5 kΩ-cm, depending on the sample size) indicate a high permeability of chlorine ions. As can be seen in Figure 5, with the progress of time and the continuation of the process of wetting and drying in the salt solution, the electrical resistance increases. After a year, the initial value more than doubled in the dry samples (from 6.4 to 16.9 kΩ-cm) and in the samples that underwent wetting and drying cycles (from 5.3 to 11.23 kΩ-cm).

#### 4.1.3. Electrochemical Behavior

**Figure 6:**
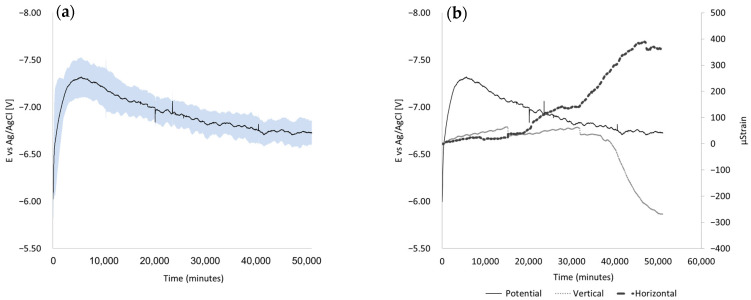
(**a**) Electrochemical behavior measurements in a 3-electrode system with 9 V external voltage: average of 3 samples (black line) and ± standard deviation (blue color); (**b**) Strain in concrete due to corrosion of reinforcing bars.

During the voltage/chloride application on the samples without the constant axial load, the electric potential was measured using a system of three electrodes. Figure 6a displays the average potential against a reference electrode for three samples (indicated by the black line in the figure), along with the corresponding standard deviation (indicated by the blue color). All results were obtained following the activation of an external electrical voltage of 9 V. Despite maintaining a constant electric voltage of 9 V, the reinforcement bars displayed a delayed response, necessitating approximately 3.5 days to achieve a maximum value of −7.3 V. Although some time elapsed before reaching the maximum, the immediate response upon voltage application was an average electric potential of −6 V. After reaching the maximum, a decrease in the electrical voltage occurred. During open-circuit conditions (without an external voltage), the average voltage readings ranged between 0.3 and 0.45 V. Upon the application of the electrical voltage, the corrosion process of the reinforcement bars commenced. This process resulted in a transformation in the volume of corrosion products, consequently giving rise to internal pressure. This internal pressure, in turn, prompted a modification in the strain occurring at the center of the concrete sample. The outcomes of these occurrences are visually depicted in Figure 6b. It is evident that the influence of corrosion products commenced altering the horizontal strain (as inferred from the curve’s slope) after 25 days (36,000 min). Similarly, a noticeable impact on the vertical strain manifested after 22 days (31,680 min). Notably, the temporal disparity between these occurrences is negligible, as the prevailing phenomenon pertains to the gradual filling of voids and the subsequent deterioration following a crack—marked by an instantaneous decline in the graph.

**Figure 7:**
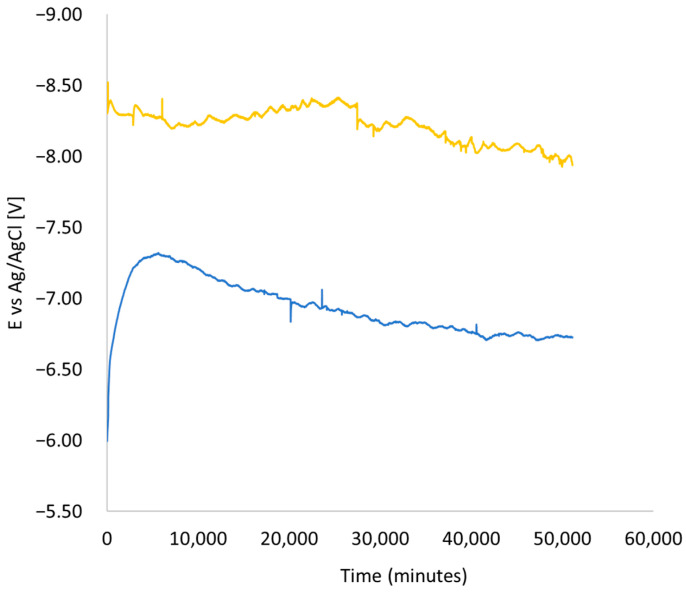
Comparison between columns affected by a voltage of 9 V (blue line) and samples that have undergone a combined impact, including a service load of 120 tons and an electrical voltage of 9 V (yellow line).

Figure 7 illustrates a comparison between the results of samples subjected to an electric voltage of 9 V, represented in blue, and the average of samples exposed to 9 V in conjunction with a service load of 1200 kN (yellow line). It is evident that the behavior of the samples remains relatively consistent and closer to the externally applied voltage of 9 V. This behavior can be attributed to the development of micro-cracks when the load is applied, facilitating the penetration of the electrolyte permeability (salt solution) deep into the sample, thereby closing the electrical circuit. After 19 days (27,360 min), the measurements indicated a slight change in the slope direction. This may indicate that the cracks are filled by corrosion products and thus make it difficult for ions to pass through this coating.

### 4.2. Reinforced Concrete Disc Specimens

The following sections describe the effects of the different variables on the electric potential and resistance, and chloride permeability of the reinforced concrete disc specimens.

#### 4.2.1. Effect of Concrete Cover Thickness and Surface Area of Steel Bars

##### Half-Cell Potential of Reinforcing Steel

**Figure 8:**
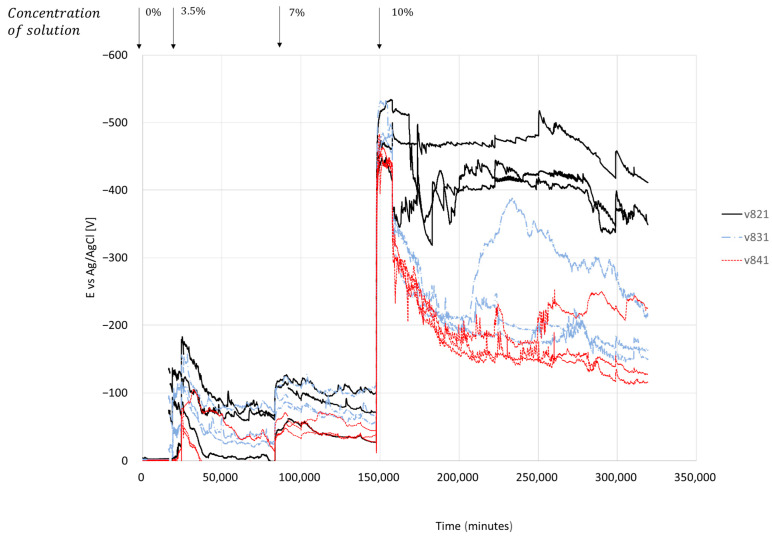
Electric potential change due to chloride concentration change, 8 mm diameter rods.

Figure 8 shows the measurement results of electric potential in the reinforcement bars located in the concrete following a change in the concentration of the exposure solution. Each change in chloride concentration elicited an immediate response in the electric potential, with discernible peaks evident in the curves (indicated by black arrows). The marking V821, V831, and V841 are for the samples with a cover thickness of 20, 30, and 40 mm, respectively, and reinforcement bars with a diameter of 8 mm. Open-circuit potential values in chloride-free solution were around −1.5 mV. After replacing chloride-free water with a 3.5% chloride solution, the average electrical potential dropped to −160.9, −125.9, and −74.4 mV for cover thicknesses of 20, 30, and 40 mm, respectively. The process of decreasing electrical potential persisted for 3.8, 6.8, and 6.4 h, respectively, until reaching local minima values. Subsequently, there was a recovery (back to more positive/noble values) of +82.7, +80.2, and +62.18 mV, respectively. The duration of recovery lasted 17.6, 18.2, and 18.8 h, respectively. After about 60 days, the salt concentration in the solution rose to 7% by weight. The observed reaction was again immediate, but less negative compared to the reaction to a previous concentration of 3.5%. Electric potential decreased within 4.8, 5.8, and 7.9 h, respectively, while the potential dropped to average values of −108.3, −105.6 and −56.7 mV, respectively. After this stage, no recovery (potential increase) was observed. The changes in electric voltage of half-cell after the concentration of chlorides was increased to 10% were −425.7, −419.1, and −425.7 mV, respectively, in 6.8, 4.6, and 3.3 h. After 218 days, the experiment was stopped. At this time, the electrical potential of half a cell showed a recovery in samples with a cover thickness of 30 and 40 mm. The values measured at this point were −176.5 and −158.2 mV for the samples with a cover thickness of 30 and 40 mm, respectively. On the other hand, the samples with a cover thickness of 20 mm exhibited values of −377 mV, suggesting ongoing corrosion with no sign of recovery for these samples.

**Figure 9:**
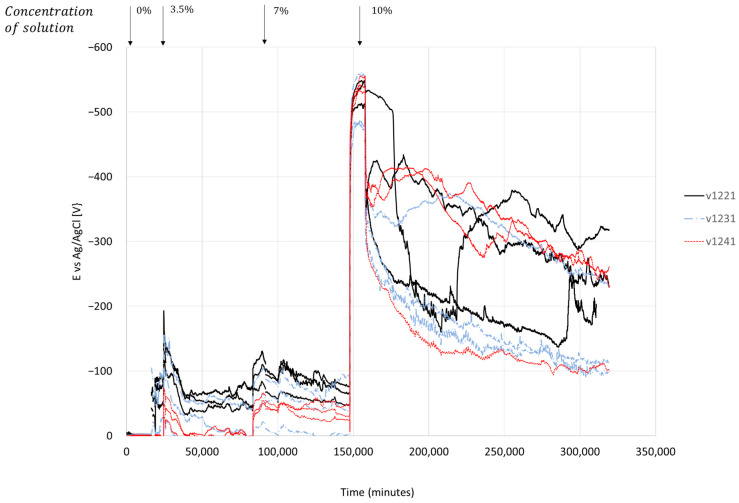
Electric potential change due to chloride concentration change, 12 mm diameter rods.

Figure 9, similar to Figure 8, illustrates the electric potential measurements of 12 mm-diameter reinforcement bars located in concrete. The samples, labeled V1221, V1231, and V1241, had cover thicknesses of 20, 30, and 40 mm, respectively. Initially, the open-circuit potential values in a chloride-free solution were approximately −3.4 mV. Subsequently, the chloride-free water was replaced with a 3.5% chloride solution, resulting in a significant decrease in electrical potential. The potential dropped to −141.7, −87.2, and −45.2 mV, respectively, for cover thicknesses of 20, 30, and 40 mm. This decrease in electric potential continued for 6, 11.2, and 5.3 h, respectively, until reaching local minima values. After the decrease in electrical potential, a recovery phase was observed, with values of +73.0, +71.0, and +40.6 mV, respectively, for cover thicknesses of 20, 30, and 40 mm, respectively. The recovery process lasted for 11.2, 38.1, and 38.7 h, respectively. After changing the salt concentration in the solution to 7% by weight, the observed response was immediate, and the electric potential decreased within 6.8, 9.6, and 7.3 h, respectively, for the different cover thicknesses. The potential values decreased to −48.6, −40.5, and −46.8 mV, respectively. Following this change, the recovery process continued until the solution concentration was further increased to 10%. At that point, the measured change in potential was +38.61, +14.90, and +23.59 mV, respectively, for the different cover thicknesses. After increasing the concentration to 10%, the measured half-cell potentials were −470.7 mV, −462.0 mV, and −511.9 mV, respectively, with a duration of the decrease lasting 7.0, 7.5, and 7.5 h, respectively, from the time of increasing chloride concentration in the solution. At the end of the experimental period, an increase in half-cell potential values was observed, with readings of +289.93, +356.39, and +340.68 mV, respectively.

##### Chloride Permeability by Colorimetric Method

**Figure 10:**
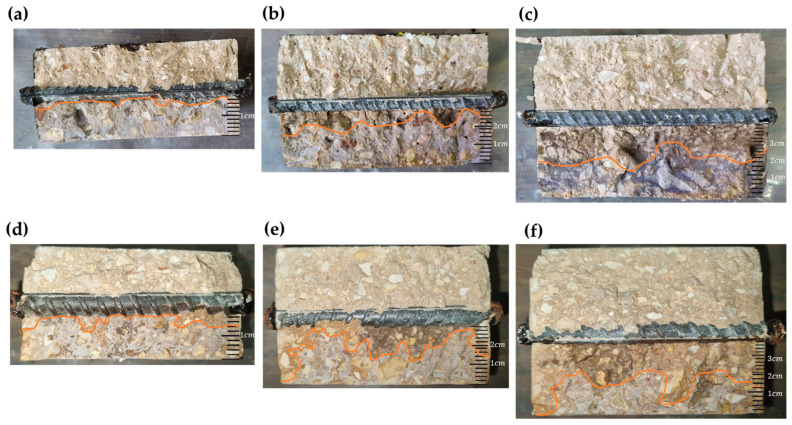
Results of AgNO_3_ reaction with chloride ions in fresh cross-sections of reinforced concrete samples containing steel bars of 8 mm (**a**–**c**) and 12 mm (**d**–**f**) diameter.

The results of the reaction of AgNO_3_ with chloride ions on a fresh cross-section of reinforced concrete samples containing rebars with diameters of 8 and 12 mm are illustrated in Figure 10. The orange line represents the boundary of free chlorides reacting with AgNO_3_. Figure 10a shows that the chlorides reached 59% of the bar interface length in the sample with a 20 mm cover thickness. In contrast, in the sample with a 30 mm cover thickness and an 8 mm bar diameter, the interface with chloride drops to only 13% of the total interface length. Lastly, in the sample with a cover thickness of 40 mm and an 8 mm bar diameter, the free chloride ions do not reach the bar at all.

In the samples with 12 mm diameter bars, the results were as follows: for a cover thickness of 20 mm, the chlorides extended 72% of the bar interface length; for a cover thickness of 20 mm, the chlorides reached 16% of the bar interface length; and for 40 mm cover thickness, there was no contact with chlorides at all, as indicated by the AgNO_3_ test.

##### Determination of Chloride Ion Concentration by Titration

To test the concentration of chlorides in the concrete and build a concentration profile, a test was performed by titration. In contrast to the traditional approach, the chlorides in the solution that was in contact with the surface increased over time, as described earlier, according to the following concentrations: 0%, 3.5%, 7%, and 10%. After disconnecting the samples from the electrical voltage measurement system, it was found that the chloride concentration on the surface of the samples was 0.3% with a precision of ± 0.022% of the weight of the concrete sample. It should be noted that according to the Energy-Dispersive Spectroscopy (EDS) results described below, this amount cannot be attributed to the cement in the mixture, as is usually the case. When entering the depth of the samples in which contact of chlorine with the reinforcing bar was not observed, according to Figure 10, decreasing values were obtained up to the level of 0.11 ± 0.06%. On the other hand, considering the 20 mm cover thickness, in the samples where contact of chlorides with the reinforcement bar was observed, the trend was different. In the center of the depth, there was no significant change in the chloride content compared to the surface, but at the bar-concrete interface, the values reached 0.55 ± 0.049%. These results were also confirmed by EDS analysis.

##### X-ray Diffraction Investigation

**Figure 11:**
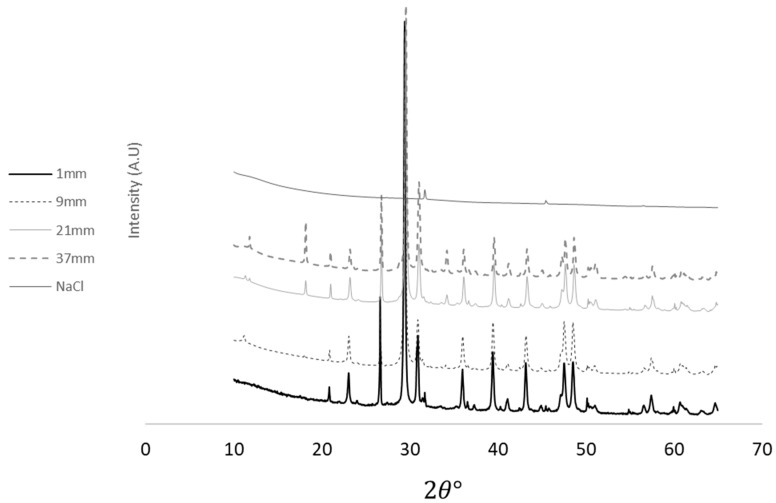
X-ray diffraction patterns of the sample materials.

The results of the study of the reaction products of the NaCl solution with the concrete surface using X-ray diffraction (XRD) are shown in Figure 11 and Table 2 for sample V821, as they did not show recovery based on the results of half-cell potential. It should be noted that in the 1 mm deep assessment, the presence of 0.8% pure NaCl is evident. For deeper samples, this value decreases to 0.1%, reaching the sensitivity threshold of the device. As expected, when this value decreases, reaction products start to appear with hydration products such as Friedel’s salt (Hydrocalumite). These products were not observed at a depth of 37 mm, which is attributed to the lack of chlorine ions in the layer of the tested sample. It is important to note that most (over 90%) of the sample contains fillers that are inert to the reaction with NaCl. Based on this, it is given that the rest of the chlorine ions do not pass through a filler and usually pass and are wiped by a limiter in a cement matrix.

##### Analysis by EDS to Detect Chlorine Ions

**Figure 12:**
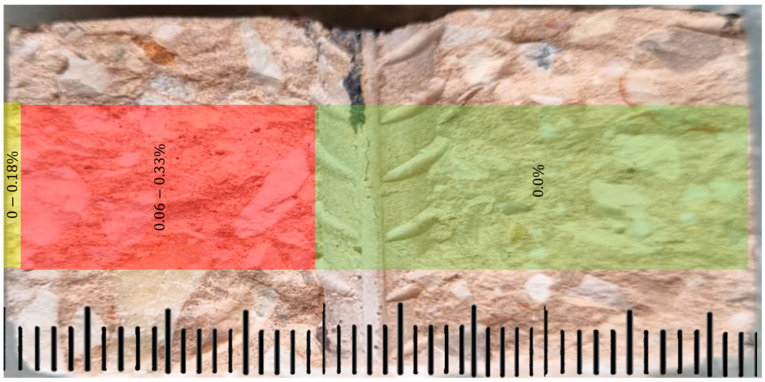
Cross section of the sample V821 analyzed in EDS.

Three prominent areas in the EDS test were observed on sample V821, as follows (Figure 12): The first area is between 0 and 1 mm depth (indicated by yellow color—Figure 12), the second section was between 1 and 20 mm (indicated by red color), and finally—the section beyond 20 mm to the end of the sample (indicated by green color). The concentration of chlorine ions detected in the test within the green area was close to zero. In the intermediate area, the median ranged from 0.06 to 0.33% by weight, with higher values scattered and making it difficult to define a position closer to the edge of the sample. In the yellow area, the concentration ranged from 0 to 0.18%. The scale division marked on the figure indicates one millimeter. The region exposed to the salt solution was located outside of the yellow area.

## 5. Discussion

**Figure 13:**
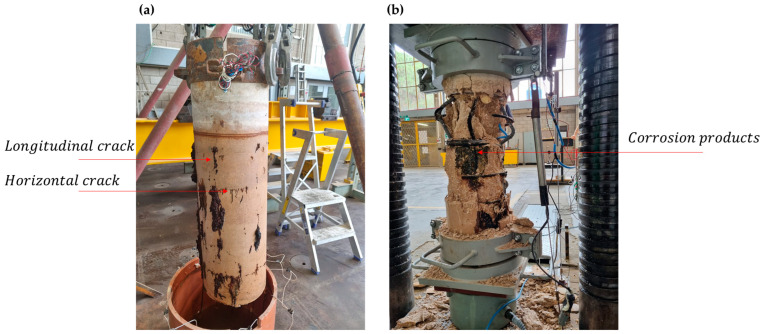
Appearance of a column specimen after 9 V exposure and chloride environment before (**a**) and after (**b**) application of compressive load.

Examining the results obtained in the salt solution exposure experiment (see Section Half-Cell Potential of Reinforcing Steel, Figure 8 and Figure 9), it can be seen that the electrochemical voltage measured in a severe case of exposure to the highly concentrated solution (10%) reaches 0.6 V. To avoid corrosion, as mentioned earlier, a voltage deviation of 0.2 V must be prevented [51]. The regulations and guidelines assume that stray currents for human safety can range between 60 and 90 V [52,53], which will inevitably lead to structural degradation of reinforced concrete structures. The damage to the structures can be attributed to the degradation observed in the column specimens’ experiments. Corrosion products introduced internal stresses and created cracks in the concrete cover (Figure 13a), which divided the cross-section into two separate parts. The external part consisted of the concrete cover shell, while the internal part was the concrete core bounded by the transverse steel reinforcement (Figure 13b).

The decrease in the strength of the samples can be attributed to two main phenomena: corrosion and lateral cracking. The corrosion of the steel reinforcement in the concrete leads to the formation of transverse and longitudinal cracks that are parallel to the reinforcement bars. Similar results were also obtained in the studies [54] and [19]. Unlike the results obtained in the studies [19,43], which showed a decrease of 47–75% in the strength of the unloaded specimens, the specimens in the present study demonstrated an average decrease of 11.85% only. Compared to the reference specimen, the deformations overall decreased. However, there was an increase in deformation due to the formation of lateral cracks. These cracks allowed the element to deform without the resistance normally provided by the full cross-section (Figure 4 and Figure 13).

These differences can also be attributed to the process of corrosion and its measurement. In the works mentioned before [19,43], the measurements were based on the Faraday law. It means corrosion development was a direct function of the corrosion current and spread uniformly along the reinforcement bars. In contrast, corrosion in the longitudinal bars in the present study was not uniform (Figure 13). The outer side of the bar (that was close to the element surface) underwent a faster corrosion process than its inner side, which did not undergo a corrosion process at all. Therefore, even a definition of corrosion current cannot be considered adequate here because not all the surfaces of the bars underwent corrosion. Another critical parameter that demands consideration is the constant applied load, which not only influences the initiation of cracks in the structural element but also exerts a notable influence on its transitional properties, as corroborated by prior observations [8].

In addition to the influence of the constant compressive axial load applied to the element, as observed in this study, the presence of reinforcement bars exacerbates the presence of chloride ions at the concrete-bar interface. A similar result was found in [8]. According to the results described in Figure 5 and Figure 6, for a larger bar diameter (12 mm compared to 8 mm), the electrical potential of a half-cell increases by 10.6%, 10.2%, and 20.2% depending on the thickness of the concrete cover (20, 30, 40 mm). The EDS results support this observation. In the area of the reinforcement bar, 0.33% chloride concentration was found in relation to the tested area.

These effects can be explained by the increase in the surface area of the rebar available for electrochemical reactions, which reduces the impact of already-formed corrosion products and creates a protective layer similar to a double layer in electrochemistry. The double layer of ions forms from two thin layers of ions at the concrete-bar interface, reducing the effect of the previously formed corrosion products. In reinforced concrete, the double layer ion consists of two thin layers of ions formed at the concrete-bar interface between the steel bar and the concrete. This double layer comprises an inner layer of negative ions and an outer layer of positive ions [55,56]. The double layer is a result of the difference in electrical potential between the steel bar and the concrete. The steel bar is more electronegative than the concrete, so it attracts positive ions from the concrete. This layer is balanced by a layer of negative ions from the concrete. The double layer plays an important role in protecting the steel bar from corrosion. The negative ions in the inner layer of the double layer repel chloride ions, which are the main cause of steel bar corrosion. The double layer also helps to maintain a high pH at the surface of the steel bar, which also helps to protect it from corrosion. Strengthening this claim, a decrease in the half-cell potential can be observed in specimens that have undergone wetting and drying cycles (Figure 5). When surface electrical resistance increases despite exposure to a salt solution, the electrical potential of the half-cell decreases at the same time. We assume that this happens due to the corrosion products surrounding the reinforcement bar, when a layer impermeable to chlorine ions is created, thus forming a secondary double layer. As depicted in Figure 9, when a load is applied and the first cracks appear, this layer is not easily formed. Therefore, the curve of the half-cell potential remains relatively more or less flat compared to the curve of the free-load specimen. The effect of a double layer is noticeable in the initial stage after applying the electric voltage, reaching a local maximum, which can be linked to the decrease in the formation of corrosion products. Despite the reduction mechanisms of a double layer, the alkalinity of the concrete, and a salt solution attack, the pH measured was ~13 throughout the experiment. As mentioned earlier, the electrical voltage to the ground usually varies between 60 and 90 V. These are very high values in relation to a 10% solution, which would give the chloride content in the concrete of about 0.33% at the concrete-bar interface because such a chloride concentration produced a maximum electrical voltage of 0.51–0.62 V. This environmental condition can be regarded as the most severe among those outlined in the existing standards [57].

Cracks in concrete are formed due to the increase in the volume of corrosion products [58] and affect two main aspects. Firstly, the mechanical behavior is impacted, resulting in significant deformations (Figure 4 and Figure 7) and crack openings (Figure 13). Secondly, the integrity of the concrete itself is compromised. In a traditional approach, increasing the thickness of the cover is thought to delay the penetration of harmful agents. However, the current work clearly demonstrates that cover thickness does affect the decay of the electric voltage when the concentration of the solution changes, as shown in the experiment performed on the disk samples. On the other hand, an increase in concrete cover thickness does not result in an increase in the electric voltage at that moment (Figure 8 and Figure 9). Therefore, it can be inferred that concrete possesses good electrical properties. Based on this, it can be concluded that an increase in the thickness of the concrete cover may not provide reliable protection against stray currents. Stray currents flowing through concrete can cause the decomposition of Friedel’s salts, which are compounds that bind chloride ions in the cement matrix. This releases the chloride ions that were previously held by the calcium silicate hydrate (C-S-H) gel, the main binding phase of cement. The released chloride ions will migrate towards the steel reinforcement, increasing the risk of corrosion. This also reduces the electrical resistance of the concrete cover, which is a measure of its durability [59].

## 6. Conclusions

In this research work, experiments were performed to examine the combined effects of compressive axial load and environmental factors (chloride attack and stray current) on the degradation of reinforced concrete. Various methodologies were used to assess the influence of concrete cover thickness and the surface area of reinforcement steel bars on the chloride concentration. The study focused on the effect of chlorides at the interface between concrete and the reinforcement bars. The primary conclusions are summarized as follows:

- The stray current affects the corrosion process, leading to a reduction in the structural properties of reinforced concrete columns in terms of strength and ductility.
- An electrical voltage of 9 V, simulating stray voltage, and an aggressive chloride environment were applied to test samples, resulting in an 11.85% decrease in bearing capacity and a 12.8% reduction in ductility (from μ = 1.94 to μ = 1.69). Further testing under a service load of 1200 kN showed more pronounced effects, with a 34.7% decrease in bearing capacity and a 41.7% reduction in ductility.
- For a combination of constant axial load alongside environmental conditions involving stray currents and chloride attacks, a reduction in the column’s maximum load-carrying capacity is observed. The reduction is less pronounced in terms of ductility (compared to the unloaded specimens). These results are attributed to the formation of early cracks and their subsequent closure upon the application of compressive axial loading.
- The amount of steel in the concrete and the surface area of the steel bar contribute to a higher concentration of chloride ions in the area at the interface between the concrete and the bar. It increases the electrical conductivity of the reinforced concrete element. This process accelerates the rate of corrosion while creating chemical bonds with the corrosion products and chlorine ions. Reinforced concrete samples with 12 mm diameter bars exhibit a 22% higher chloride penetration at the interface compared to samples with 8 mm diameter bars, with a cover thickness of 20 mm, as indicated by the results of the AgNO_3_ test.
- An electrochemical reaction initiates at the concrete-bar interface after a change in chloride concentration across the specimen surface. Consequently, under moist conditions, the onset of corrosion is not influenced by permeability time.
- Under the given conditions, no influence of the concrete cover thickness on the electrochemical reactions of the reinforcement bars inside the concrete was observed. Consequently, based on these findings, it does not provide adequate protection against stray currents.
- In future work, the aim is to investigate stray currents in a realistic scenario and to evaluate an existing model that can predict the service life of buildings near railway tracks.

## Figures and Tables

**Table 1:** The concrete mixes.

Content (kg/m^3^)						W/C ^3^	Air Content (%)	Unit Weight (kg/m^3^)	Slump ^4^ (mm)
C ^1^	W ^1^	S ^1^	Aggregates ^2^						
			Fine	Coarse	Maximum Aggregate Size				
					(mm)				
500	195	5	1100	500	9	0.39	1.9	2281	S5 (138)

^1^ C—Cement; W—Water; S—Superplasticizers. ^2^ Type of Aggregates—coarse: limestone, fine: quartz. ^3^ W/C—water-to-cement ratio. ^4^ Based on the IS 26 standard (Testing concrete: Fresh concrete—Consistency—Slump test [36]).

**Table 2:** XRD profile analysis at various depths of the sample.

Chemical Component		Sample Distance from the Surface (mm)			
		1	9	21	37
		%Content			
Calicite	$\mathrm{Ca}\left({\mathrm{CO}}_{3}\right)$	76.7	82.2	71.9	58.1
Dolomite	$\mathrm{CaMg}{\left({\mathrm{CO}}_{3}\right)}_{2}$	14.3	10.9	15.9	22.7
Quartz	${\mathrm{SiO}}_{2}$	8.2	5.3	8	9.2
Halite	NaCl	0.8	0.1	0.1	0.1
Portlandite	Ca(OH)_2_		0.5	2.5	6.2
Hydrocalumite	${\mathrm{Ca}}_{4}{\mathrm{Al}}_{2}{\left(\mathrm{OH}\right)}_{12}{\left(\mathrm{Cl},{\mathrm{Co}}_{3},\mathrm{OH}\right)}_{2}\cdot 4H_{2}O$	-	0.1	0.3	-
Forsterite	$\mathrm{Mg}2\left({\mathrm{SiO}}_{4}\right)$		0.9	0.6	0.8
Tetracalcium Dialuminium Dodecahydroxide Carbonate Pentahydrate	$3\mathrm{CaO}\cdot {\mathrm{Al}}_{2}O_{3}\cdot {\mathrm{CaCO}}_{3}\cdot 11H_{2}O$		-	0.7	2.3
Vesuvianite	$\left(\mathrm{Ca},\mathrm{Na}\right)19\left(\mathrm{Al},\mathrm{Mg},\mathrm{Fe}\right)13\left({\mathrm{SiO}}_{4}\right)10\left({\mathrm{Si}}_{2}O_{7}\right)4\left(\mathrm{OH},F,O\right)10$		-	-	0.6

## Data Availability

The data presented in this study are available on request from the corresponding author.
